# Long Noncoding RNA 6302 Regulates Chicken Preadipocyte Differentiation by Targeting *SLC22A16*

**DOI:** 10.3390/genes15060758

**Published:** 2024-06-09

**Authors:** Xiangfei Ma, Yuehua He, Cong Liu, Tingqi Zhu, Donghua Li, Wenting Li, Guirong Sun, Xiangtao Kang

**Affiliations:** 1 College of Animal Science and Technology, Henan Agricultural University, Zhengzhou 450002, China; mxf1228@126.com (X.M.); 18438405655@163.com (Y.H.); liucong8706@126.com (C.L.); ztq560713@163.com (T.Z.); lidonghua6656@126.com (D.L.); liwenting@henau.edu.cn (W.L.); 2 The Shennong Laboratory, Zhengzhou 450002, China

**Keywords:** *SLC22A16*, lncRNA, chicken, abdominal adipocytes

## Abstract

The excessive deposition of abdominal adipocytes in chickens is detrimental to poultry production. However, the regulatory factors that affect abdominal adipogenesis in chickens are still poorly understood. *SLC22A16* is differentially expressed in abdominal preadipocytes and 10-day differentiated adipocytes in chickens, but its role in regulating chicken adipogenesis has not been reported. In this study, the function of *SLC22A16* in chicken abdominal preadipocytes was investigated. *SLC22A16* is significantly upregulated during abdominal adipocyte differentiation. The overexpression of *SLC2A16* upregulated the expression of adipogenic marker genes and proliferation-related genes, and promoted the proliferation of adipocytes and the accumulation of triglycerides. The knockdown of *SLC22A16* downregulated the expression of adipogenic marker genes and proliferation-related genes, inhibited the proliferation of adipocytes, and impaired the accumulation of triglycerides in adipocytes. In addition, *LNC6302* was differentially expressed in abdominal preadipocytes and mature adipocytes, and was significantly positively correlated with the expression of *SLC22A16*. Interference with *LNC6302* inhibits the expression of adipogenic marker genes and proliferation-related genes. The data supported the notion that *LNC6302* promotes the differentiation of chicken abdominal adipocytes by cis-regulating the expression of *SLC22A16*. This study identified the role of *SLC22A16* in the differentiation and proliferation of chicken adipocytes, providing a potential target for improving abdominal adipogenesis in chickens.

## 1. Introduction

In the poultry industry, abdominal fat is a component of the chicken body, and the excessive accumulation of abdominal fat can affect feed efficiency, meat quality, and consumer preferences [1,2]. Therefore, the accumulation of fat in the chicken abdomen is a disadvantageous factor for both merchants and consumers [3,4,5]. It was reported that the heritability of abdominal fat (0.82) was significantly higher than that of chest muscle (0.55) and body weight (0.55) [6], and that chicken abdominal preadipocytes have higher adipogenic differentiation ability than intramuscular preadipocytes [7]. Lipogenesis is regulated by a series of key transcription factors, such as peroxisome proliferator-activated receptor γ (*PPARγ*), the CCAAT/enhancer-binding protein α (*C/EBPα*), and fatty acid binding protein 4 (*FABP4*) [8,9]. Although there are currently many research reports on the deposition of abdominal fat in chickens, adipocyte differentiation is a complex and delicate process, and there are still many potential targets that have not been discovered. Therefore, it is important to study the molecular mechanisms of adipogenesis, which can accelerate the improvement of excessive abdominal fat in chicken.

At present, multiple genomic approaches can offer methods for identifying the genes or molecules responsible for fat deposition. For example, mRNA transcriptomics was used to identify 1700 differentially expressed mRNAs in the intramuscular fat of 24-month-old bulls and calves in Qinchuan cattle [10]. Moreover, 6814 differentially expressed genes were screened in goat subcutaneous preadipocytes and mature adipocytes using RNA-seq [11]. Long noncoding RNAs (lncRNAs) are a class of RNA with a length greater than 200nt and no or weak protein-coding potential, which regulates many levels of gene regulation from transcription to translation [12]. An increasing number of lncRNAs are being identified in fat metabolism and differentiation processes; for example, 283 differentially expressed lncRNAs were identified in preadipocytes and 3T3L1 adipocytes differentiated for 12 days [13]. A total of 2135 lncRNAs were identified during the differentiation of intramuscular adipocytes in the longissimus dorsi and semitendinosus of pigs [14]. The mechanism of lncRNAs’ function is related to their sequence, structure, and subcellular localization. lncRNAs in the nucleus regulate transcription and modify newly formed RNA through chromatin interactions and remodeling [15,16]. lncRNAs in the cytoplasm can regulate translational programs and assist in protein processing [17,18,19]. Recently, some lncRNAs related to fat formation have been identified. The knockdown of *lncRNA NR_015556* upregulates *PPARγ* and *C/EBPα* to promote C3H10T1/2 cell differentiation [20]. The knockdown of lncRNA *MIR1HG* reduces the enrichment of acetylation (AcH3) and histone H3 lysine 4 trimethylation (H3K4me3) in the *FABP4* promoter, thereby inhibiting the adipocyte differentiation of human adipose-derived stem cells [21]. *AdipoQ* antisense lncRNA (AdipoQ AS lncRNA) inhibits mouse adipogenesis by forming an *AdipoQ* AS lncRNA/AdipoQ mRNA duplex [22]. *LncPLAAT3-AS* upregulates *PLAAT3* expression by absorbing miR-503-5p to promote the differentiation of porcine preadipocytes [23]. These reports support the importance of both coding genes or lncRNAs in the process of adipogenesis.

This study found the differential expression of *SLC22A16* in chicken abdominal preadipocyte and differentiated adipocytes for 10 days [24], predicting its involvement in regulating the generation of chicken adipocytes. *SLC22A16* is a member of the Solute Carrier protein (SLC) family, which includes membrane-bound transport proteins that manage multiple substrates and regulate the homeostasis of endogenous metabolites. At present, the reports on *SLC22A16* only involve the pharmacokinetics of doxorubicin and carnitine transport, and there are no studies reporting the role of *SLC22A16* in adipose differentiation [4,25,26,27]. To analyze the functionality of *SLC22A16*, we investigated the effect of *SLC22A16* on the proliferation and differentiation of chicken abdominal adipocytes through overexpression and interference techniques. The results showed that *SLC22A16* promotes the proliferation and differentiation of chicken abdominal adipocytes. Through the joint analysis of lncRNA data differentially expressed in chicken abdominal preadipocytes and adipocytes, a significant correlation was found between *LNC6302* and *SLC22A16* expression. *LNC6302* promotes the proliferation and differentiation of chicken abdominal adipocytes by regulating the expression of *SLC22A16*. In summary, our study provides a potential target for improving abdominal fat deposition in chickens.

## 2. Materials and Methods

### 2.1. Ethics Statement

All animal experiments were approved by the Animal Care Committee of the College of Animal Science and Technology, Henan Agricultural University (approval code HNND2022030840; 7 March 2022), and were performed following the protocol approved by the Institutional Animal Care and Use Committee (IACUC) of China. All efforts were made to minimize animal suffering.

### 2.2. Cell Culture

Primary chicken abdominal preadipocytes were isolated from the abdominal adipose tissue of two-week-old chickens following the methods described previously [28]. The IPC1 (immortal chicken abdominal preadipocytes infected with TERT retrovirus) preadipocyte strain was donated by Northeast Agricultural University [29]. IPC1 preadipocytes or primary abdominal preadipocytes were seeded in 12-well plates and maintained with basic medium (DMEM/F12, 10% FBS, and 1% penicillin/streptomycin). Once the cells reached 90% confluence, the differentiation medium (0.5 mM 3-isobutyl-1-methylxanthine, 1 µM dexamethasone, 10 µg/mL insulin, and 300 µM oleic acid) was used to replace the basic medium for 48 h. The differentiation medium was then replaced with maintenance medium (10 µg/mL insulin and 300 µM oleic acid) and maintained for 6 days.

### 2.3. Oil Red O Staining and Cellular TG Content Measurement

The cells to be tested were collected, washed with PBS three times, and fixed with 4% paraformaldehyde for 30 min. After that, the cells were washed twice with PBS and then stained with Oil Red O solution (60% Oil Red O, 40% deionized water) for 20 min. Before imaging, the cells were washed with PBS three times. All procedures were performed at room temperature. TG content was measured using a triglyceride content detection kit (APPLYGEN, Beijing, China) according to the manufacturer’s instructions. Briefly, lysis buffer was added to the cell precipitate, which was incubated at room temperature for 10 min; the cell supernatant was heated at 70 °C for 10 min, and then centrifuged at 2000 rpm for 5 min, with the supernatant then used for the assay.

### 2.4. Rapid Amplification of cDNA Ends (RACE)

In order to obtain the full-length sequence of *LNC6302*, we performed a RACE assay using the SMARTer RACE cDNA 5′/3′Kit (Clontech, Palo Alto, CA, USA) according to the manufacturer’s instructions. The total RNA isolated from preadipocytes and adipocytes was mixed and used for the RACE experiment. The primers used for 5′ and 3′ RACE were 5′-AAGTGTTAGTCAAAGGTTTTCCAA-3′ and 5′-CAGCATTAGAAAAGATAGAGATGT-3′, respectively.

### 2.5. Vector Construction

To construct the overexpression vectors, the chicken *SLC22A16* coding region was cloned using *SLC22A16* CDS primer. Then, the CDS fragment of *SLC22A16* was inserted into the pcDNA3.1-EGFP vector using Hind III and EcoR I sites. To verify whether *LNC6302* has the ability to encode proteins, we used a prokaryotic expression system in vitro and inserted the largest open reading frame fragment of *LNC6302* into the pET-30a vector and induced translation using an IPTG inducer. pET-30a was used as a negative control, and pET-30a-EGFP as a positive control. Then, 5× loading buffer was added to the precipitate of the induced product and incubated at 100 °C for 10 min. Finally, electrophoresis detection was performed using SDS-PAGE. The primers used are shown in Table S1. Lipofectamine 2000 (Invitrogen, Carlsbad, CA, USA) was used for plasmid transfection according to the manufacturer’s instructions.

### 2.6. RNA Interference

siRNAs specifically target *SLC22A16*, and negative control siRNAs were ordered from GenePharma (Shanghai, China). The sequences of siRNAs for *SLC22A16* were 5′-CCAGGCACACAGAACAATT-3′, and for negative control were UUCUCCGAACGUGUCACGUTT-3′. The lncRNA Smart Silencer for *LNC6302* was used for RNA interference (RiboBio, Guangzhou, China). The primary abdominal adipocytes were transfected with 50 nM lncRNA Smart Silencer supplemented with reagent buffer following the manufacturer’s instructions. The medium was changed after 4–6 h of transfection. Then, cells were collected for downstream experiments.

### 2.7. Propidium Iodide Staining and Flow Cytometry Analysis

The treated cells were digested with trypsin, washed with PBS, and then fixed in 70% ethanol at −20 °C overnight. The DNA was incubated with propidium iodide (Solarbio, Beijing, China) staining solution at 4 °C for 30 min. The cells were measured using a BD Accuri C6 flow cytometer (BD Biosciences, San Jose, CA, USA).

### 2.8. Cell Proliferation Assays

The cell proliferation analysis and cell viability assays were performed using a CCK-8 kit and EdU proliferation assay, respectively. The cells were cultured in 96-well plates and, after transfection, 10 µL of CCK-8 solution was added to the cells every 12 h according to the manufacturer’s instructions (Dojindo, Kumamoto, Japan). After incubation in a 5% CO_2_ 37 °C incubator for 2 h, the cell viability was detected at 450 nm using a Microplate Reader (Thermo, Waltham, MA, USA). The cells were cultured in 12-well plates, transfected for 48 h, and tested according to the instructions provided by the Cell-Light EdU Apollo 567 in vitro kit manufacturer (RiboBio, Guangzhou, China). Finally, the cells were photographed and recorded under a fluorescence microscope (Olympus, Tokyo, Japan) and counted using Image J software (version number: Ij53-win-java8) (NIH, Bethesda, MD, USA).

### 2.9. RNA Fluorescence In Situ Hybridization (RNA FISH) and Cytoplasmic and Nuclear RNA Extraction

FITC-labeled *LNC6302* probes for the FISH assay were synthesized from RiboBio and the FISH kit was used according to its instructions (RiboBio, Guangzhou, China). The cytoplasmic and nuclear RNA were extracted using the PARIS^TM^ Kit (Life, Boston, MA, USA) based on the manufacturer’s instructions.

### 2.10. Total RNA Isolation, cDNA Synthesis, and Real-Time Quantitative PCR (RT-qPCR)

The total RNA was extracted from the cells using TRIzol reagent according to the manufacturer’s instructions. The extracted RNA (1 µg) was converted into cDNA using the PrimeScript^TM^ RT reagent kit with Gdna Eraser (TaKaRa, Tokyo, Japan). Quantitative RT-PCR was performed using a LightCycler^®^96 system (Roche, Basel, Switzerland) and SYBR Premix Ex Taq II kit (Vazyme, Nanjing, China). *β-actin* was selected as a reference gene, and the relative quantification of genes was performed using the 2^−ΔΔCt^ method [30].

### 2.11. Statistical Analysis

All assays were performed in triplicate. For statistical analysis, all data are presented as mean ± SEM. Before applying Student’s *t*-tests using GraphPad Prism 7.0 software (San Diego, CA, USA), we used the Shapiro–Wilk method to test whether the data followed a normal distribution. A *p*-value > 0.05 indicated that the data conformed to a normal distribution. A *p*-value < 0.05 was considered statistically significant in the Student’s *t*-test.

## 3. Results

### 3.1. Differential Expression of SLC22A16 in Chicken Adipocyte Differentiation

**Figure 1:**
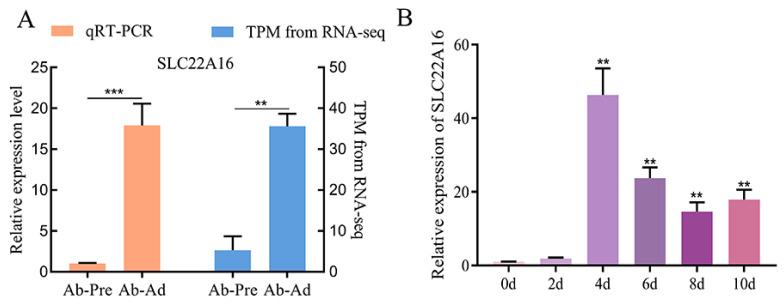
Differential expression of *SLC22A16* in chicken adipocyte differentiation. (**A**) Verification of the accuracy of sequencing results by qRT-PCR. TPM: transcripts per million. (**B**) Dynamic expression levels of *SLC22A16* during adipogenic differentiation. These data are expressed as mean ± SEM (*n*  =  3), ** *p*  <  0.01, *** *p* < 0.001.

We found the differential expression of *SLC22A16* in RNA-seq between the primary chicken abdominal preadipocytes and 10-day differentiated adipocytes. The qPCR identification of the expression of *SLC22A16* showed that it had a higher expression level than preadipocytes and showed significantly upregulated expression in 10-day differentiated adipocytes (Figure 1A) (*p* < 0.01). During the differentiation process of chicken abdominal adipocytes, *SLC22A16* showed an overall upward trend after differentiation, with the highest expression level at 4d (Figure 1B) (*p* < 0.01), suggesting that *SLC22A16* plays a catalytic role in chicken adipogenesis.

### 3.2. SLC22A16 Promotes the Differentiation of IPC1 Preadipocytes

**Figure 2:**
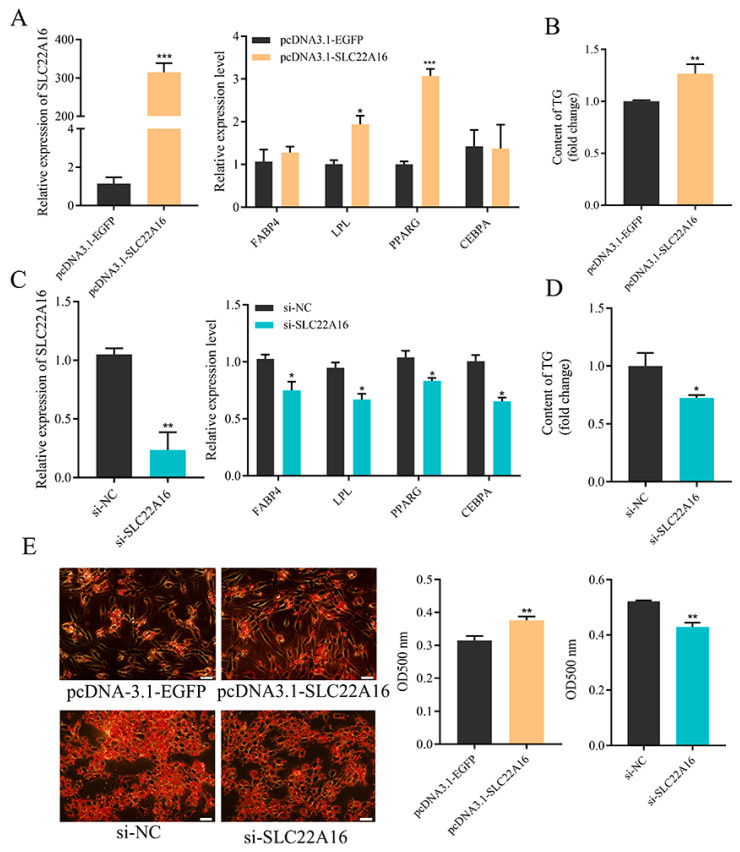
*SLC22A16* promotes the differentiation of chicken preadipocyte. (**A**) Overexpression efficiency of *SLC22A16* and changes in adipocyte differentiation marker genes after overexpression of *SLC22A16*. (**B**) Changes in intracellular triglyceride content after overexpression of *SLC22A16*. (**C**) Interference efficiency of *SLC22A16* and changes in adipocyte differentiation marker genes after interference with *SLC22A16*. (**D**) Changes in intracellular triglyceride content after interference with *SLC22A16*. (**E**) Number of lipid droplets in adipocytes stained with Oil Red O after overexpression and interference with *SLC22A16*. Scale bar: 100 µm. (mean ± SEM, *n* = 3; * *p* <  0.05, ** *p*  <  0.01, *** *p* < 0.001).

To determine the role of *SLC22A16* in preadipocyte differentiation, we used the overexpression and knockdown of *SLC22A16* in ICP1 preadipocytes. First, the overexpression of *SLC22A16* promotes the differentiation of ICP1 preadipocytes and significantly upregulates the mRNA expression of *LPL* and *PPARG* (Figure 2A) (*p* < 0.05). The intracellular triglyceride content and Oil Red O staining results showed that the overexpression of *SLC22A16* significantly increased lipid droplet accumulation (Figure 2B,E) (*p* < 0.05). Second, knockdown of *SLC22A16* significantly downregulated the mRNA levels of lipid marker genes *CEBPA, LPL*, *FABP4*, and *PPARG* (Figure 2C) (*p* < 0.05). Furthermore, evidence from the intracellular triglyceride assay and Oil Red O staining demonstrated that lipid droplet accumulation significantly declined in the *SLC22A16* knockdown group (Figure 2B,E) (*p* < 0.05).

### 3.3. SLC22A16 Promotes the Proliferation of ICP1 Preadipocytes

**Figure 3:**
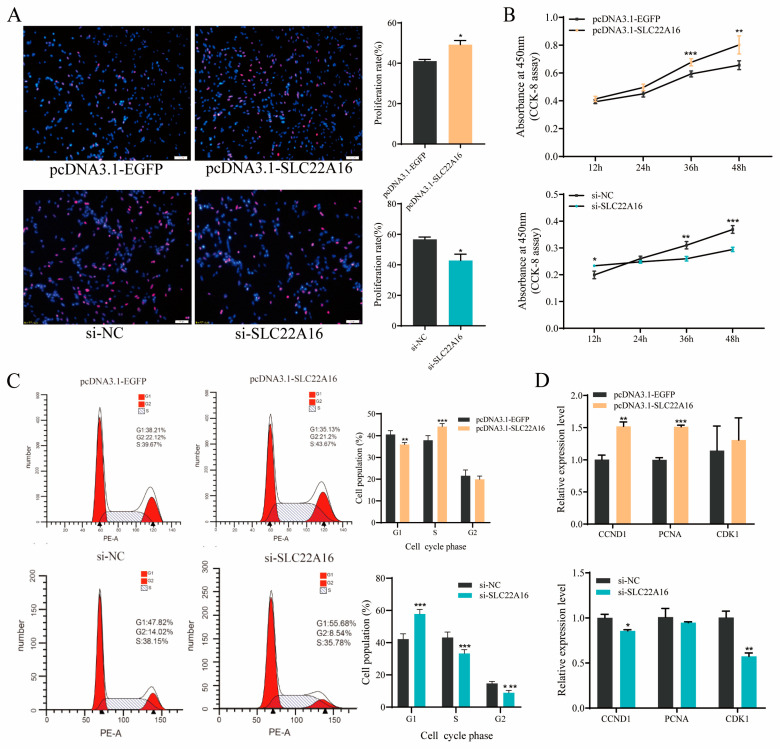
*SLC22A16* promotes the proliferation of chicken adipocytes. (**A**) EdU was used to detect the proliferation of chicken adipocytes after overexpression and interference with *SLC22A16*. Scale bar: 100 µm.(**B**) CCK-8 was used to detect cell growth after overexpression and interference with *SLC22A16*. (**C**) Cell cycle analysis of cells after overexpression and interference with *SLC22A16*. (**D**) Relative mRNA expression of proliferative genes 48 h after overexpression and interference with *SLC22A16* (mean ± SEM, *n* = 3; * *p* <  0.05, ** *p*  <  0.01, *** *p* < 0.001).

To detect whether *SLC22A16* is involved in chicken preadipocyte proliferation, we used the overexpression and knockdown of *SLC22A16* in ICP1 preadipocytes. EdU staining and a CCK-8 cell viability assay showed that the overexpression of *SLC22A16* significantly promoted the proliferation of ICP1 preadipocytes, while knocking out *SLC22A16* significantly inhibited the proliferation of ICP1 preadipocytes (Figure 3A,B) (*p* < 0.05). After *SLC22A16* was knocked out, the number of cells in G1 phase was significantly increased, and the number of cells in S phase and G2 phase was significantly reduced (Figure 3C) (*p* < 0.05). Then, G1/S-specific cyclin-D1 (*CCND1*) and proliferating cell nuclear antigen (*PCNA*) were significantly decreased after *SLC22A16* overexpression. The knockdown of *SLC22A16* significantly inhibited the mRNA expression levels of *CCND1* and *CDK1* (Figure 3D) (*p* < 0.05).

### 3.4. LNC6302 Is Associated with SLC22A16 Expression and Characterization of the LNC6302 Sequence

**Figure 4:**
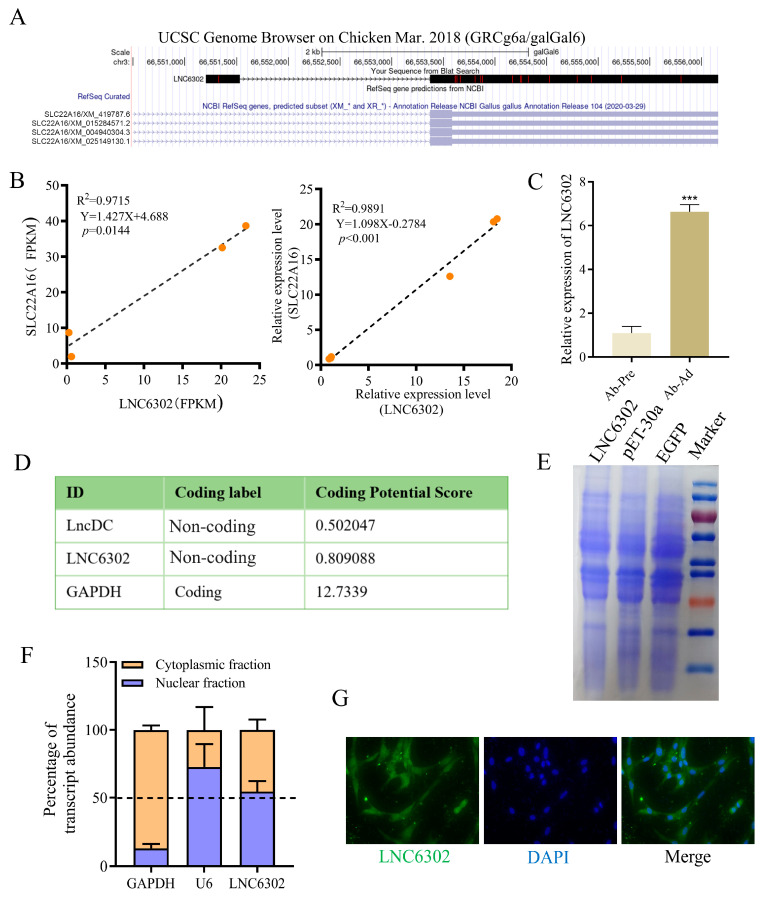
Characterization of the *LNC6302* sequence. (**A**) Genome location of chicken *LNC6302* and *SLC22A16*. (**B**) Correlation analysis of the expression of *LNC6302* and *SLC22A16* in RNA-seq (FPKM) and RT-qPCR (Ct values) data. (**C**) Relative expression levels of *LNC6302* in Ab-pre and Ab-Ad. (**D**) Coding potential of RNA sequences *LNC6302*, *lncDC*, and *GAPDH* using the Coding Potential Calculator (CPC) program. (**E**) In vitro translation assay using *LNC6302* and EGFP constructs. Shown is Coomassie Blue staining. (**F**) Expression level of *LNC6302* in nuclear and cytoplasm detected by RT-qPCR. (**G**) Location of *LNC6302* in chicken preadipocytes detected by FISH (mean ± SEM, *n* = 3; *** *p* < 0.001).

In order to understand the potential mechanism of the *SLC22A16* gene functions in chicken abdominal adipocytes, we conducted a correlation analysis between the differentially expressed mRNA and lncRNA sequencing data of abdominal preadipocytes and 10-day differentiated adipocytes in chickens and found that differentially expressed *LNC6302* was co-localized with *SLC22A16* [7,24]. To further investigate *LNC6302*, we first characterized the sequence of *LNC6302*. The RACE experiment showed that *LNC6302* had a polyadenylated transcript length of 3713bp (Figure S1). Using the BLAT tool in the UCSC Genome Browser (https://genome.ucsc.edu/ accessed on May 26, 2024) revealed that *LNC6302* is located within the *SLC22A16* gene (Figure 4A). In addition, the expression levels of *LNC6302* and *SLC22A16* were analyzed using CT and FPKM values, and the results showed a significant positive correlation between the expression levels of *LNC6302* and *SLC22A16* (Figure 4B); moreover, *LNC6302* was also significantly expressed after abdominal adipocyte differentiation (Figure 4C). The analysis results of CPC online software (https://cpc.gao-lab.org/docs/quick_guide.jsp) accessed on 18 July 2023)showed that the coding ability of *LNC6302* is similar to that of *lncDC* reported in previous studies [31], and its coding ability is much lower than that of the coding gene *GAPDH* (Figure 4D). Meanwhile, the *LNC6302* expression vector failed to produce protein through a translation assay in vitro, indicating that *LNC6302* does not have coding potential (Figure 4E). The qPCR analysis of fractionated nuclear and cytoplasmic RNA showed that *LNC6302* was expressed in both the nucleus and cytoplasm (Figure 4F). This result was confirmed by FISH (Figure 4G).

### 3.5. Interference with LNC6302 Inhibits the Differentiation and Proliferation of Abdominal Preadipocytes

**Figure 5:**
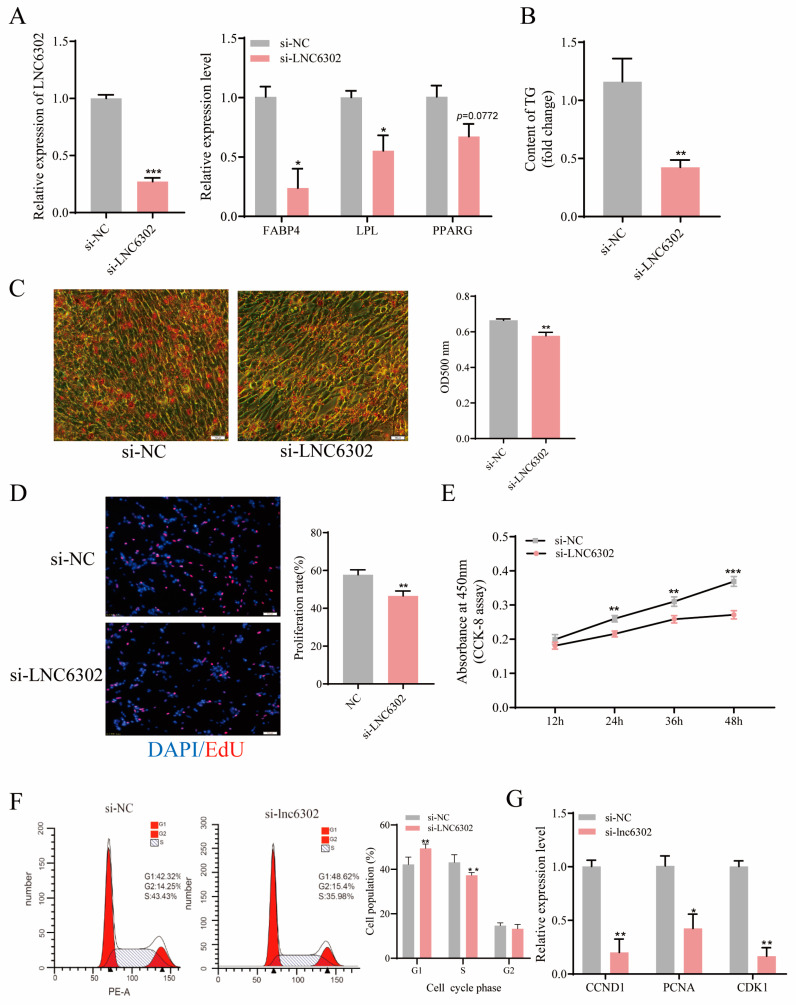
Interference with *LNC6302* inhibits the differentiation and proliferation of abdominal preadipocytes. (**A**) Relative expression level after interference with *LNC6302* and the changes in adipocyte differentiation marker genes after interference with *LNC6302*. (**B**) Changes in intracellular triglyceride content after interference with *LNC6302*. (**C**) Number of lipid droplets in adipocytes stained with Oil Red O after interference with *LNC6302*. Scale bar: 100 μm. (**D**) EdU was used to detect the proliferation of chicken adipocytes after interference with *LNC6302*. Scale bar: 100 μm. (**E**) CCK-8 was used to detect cell growth after interference with *LNC6302*. (**F**) Cell cycle analysis of cells after interference with *LNC6302*. (**G**) Relative mRNA expression of proliferative genes 48 h after interference with *LNC6302* (mean ± SEM, *n* = 3; * *p* <  0.05, ** *p*  <  0.01, *** *p* < 0.001).

To further verify the role of *LNC6302* in adipocyte differentiation, we knocked down *LNC6302* in primary abdominal preadipocytes. After knocking out *LNC6302*, the expression levels of *FABP4*, *LPL*, *PPARA*, and *PPARD* were significantly inhibited (*p* < 0.05). *PPARG* showed a downward trend (*p* = 0.0772) (Figure 5A), the intracellular triglyceride content was significantly reduced (Figure 5B) (*p* < 0.05), and the Oil Red O staining results also showed a significant decrease in lipid droplet accumulation (Figure 5C) (*p* < 0.05). Furthermore, EdU staining and the CCK8 assay suggested that the knockdown of *LNC6302* dramatically inhibited the proliferation of primary abdominal preadipocytes (Figure 5D,E) (*p* < 0.05). The flow cytometry detection results showed that knocking down *LNC6302* inhibited the proportion of S-phase cells (Figure 5F) (*p* < 0.05), and the qPCR results showed that the proliferation marker genes *CCND1*, *CDK1*, and *PCNA* were also significantly inhibited (Figure 5G) (*p* < 0.05).

### 3.6. LNC6302 Promotes Abdominal Preadipocyte Differentiation by Activating SLC22A16 in Cis-Regulating Manner

**Figure 6:**
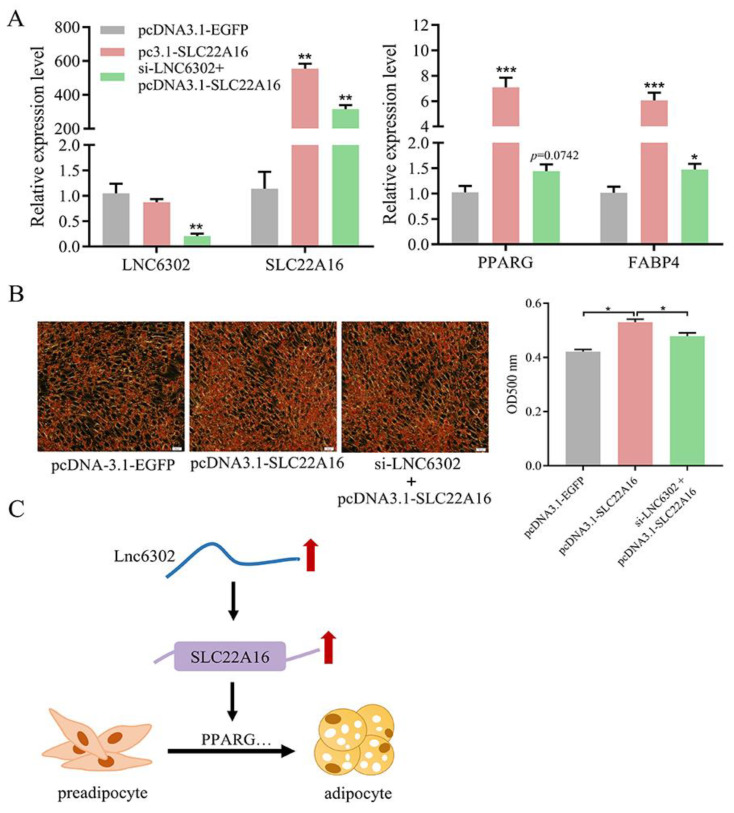
(**A**) Expression level of *LNC6302*, *SLC22A16*, and adipogenic markers *PPARG* and *FABP4* evaluated by RT-qPCR. (**B**) Oil Red O staining of abdominal preadipocytes transfected with si-NC, si-*LNC6302*, or pcDNA3.1-*SLC22A16* and si-*LNC6302*. Scale bar: 100 μm. (**C**) Proposed model of *LNC6302* regulation on adipocyte differentiation. LNC6302 carries out cis-activiting regulation of SLC22A16, which promotes targets such as PPARG, thus regulating adipogenesis. Red arrow: Upregulated expression. Black arrow: Regulatory relationship. (mean ± SEM, *n* = 3; * *p* <  0.05, ** *p*  <  0.01, *** *p* < 0.001).

To test whether *LNC6302* inhibits adipogenesis in abdominal adipocytes in a *SLC22A16*-dependent manner, si-*LNC6302* with pcDNA3.1-*SLC22A16* vector was transfected into the cells. After the overexpression of *SLC22A16*, the expression of *SLC22A16*, *PPARG*, and *FABP4* was significantly upregulated, and Oil Red O staining showed a significant increase in lipid droplet content (*p* < 0.05). When si-*LNC6302* and pcDNA3.1-*SLC22A16* co-transfection was conducted, the promoting effect of *SLC22A16* on adipocyte differentiation was inhibited by si-*LNC6302* (Figure 6A,B) (*p* < 0.05). As shown in Figure 6C, *LNC6302* promotes the differentiation of chicken abdominal adipocytes by cis-regulating the expression of *SLC22A1*6.

## 4. Discussion

Excessive fat deposition has always been a major problem faced by the poultry industry, as it poses certain obstacles to profitable agricultural economies. So far, various methods have been explored to reduce fat deposition, including feeding methods, feed formulas, and genetic selection [32,33,34]. However, adipogenesis is a delicate and complex process closely linked with the proliferation and differentiation of adipocytes, as well as the accumulation of lipids within mature adipocytes [35]. In recent years, there have been many reports about adipocyte differentiation [36,37,38]. In our previous research, we used chicken preadipocytes differentiated for 0 and 10 days to carry out RNA seq transcriptome sequencing [24]. *SLC22A16* is one of the differential genes, and *SLC22A16* is upregulated in the process of chicken adipocyte differentiation, suggesting that it may participate in adipocyte differentiation. However, the impact of *SLC22A16* on adipocyte differentiation and adipogenesis has not been reported. 

The SLC family is a member of the Major Facilitator Superfamily (MFS), which is mainly responsible for the transport of various carbohydrates, organic alcohols, and other substances in organisms [39]. Although more than 100 transporter families have been identified and classified, the SLC family is the second largest membrane protein family after G-protein-coupled receptors [40]. Therefore, the importance of the SLC transporter family to organisms is self-evident. There are reports that *SLC22A16* is mainly responsible for the transport of organic cations and carnitine [41]. Knocking down *SLC22A16* reduces the viability and cell cycle progression of acute myeloid leukemia cells, and can lead to pupal arrest in fruit flies [42,43]. The expression of *SLC22A16* in gastric cancer tissue is higher than that in normal tissue. In patients with recurrent treatment, the expression of *SLC22A16* is also significantly higher than that in patients without recurrence [44]. Iron is one of the indispensable factors in DNA synthesis in organisms [45], and iron depletion in melanoma SK-Mel-28 cells can lead to the downregulation of the polyamine import gene *SLC22A16* [46]. Atsushi Enomoto found that *SLC22A16*-mediated L-carnitine transport is essential during spermatogenesis [47], indicating that *SLC22A16* plays an important role in maintaining cell viability.

In this study, we first investigated the function of *SLC22A16* on the proliferation of chicken abdominal adipocytes by the overexpression and interference with *SLC22A16*. The EdU assay and cell cycle assay showed that the overexpression of *SLC22A16* can significantly promote the proliferation of chicken abdominal preadipocytes and significantly promote the expression of *CCND1* and *PCNA* mRNA. These two genes are used as marker genes for proliferation. *CCND1*, one of three unlinked proteins, regulates the transition between G1 and S phases of the cell cycle [48]. *PCNA* plays an essential role in the process of DNA replication and repair [49]. In addition, this study indicates that *SLC22A16* promotes the differentiation of abdominal preadipocytes in chickens. The results of the Oil Red O staining and triglyceride content assay showed a significant increase in lipid droplet accumulation in chicken abdominal adipocytes. The expression levels of *PPARG* and *CEBPA* were significantly altered by *SLC22A16*, and there is ample evidence to suggest that *PPARG* and *CEBPA* are the main transcription factors involved in adipocyte differentiation [50,51,52]. lncRNAs are important regulatory molecules and actively participate in various biological processes [53,54,55,56]. The role of lncRNAs in chicken adipocyte differentiation has also attracted the attention of researchers [57,58]. In order to understand the upstream regulatory mechanism of *SLC22A16*, this study conducted a joint analysis with our published lncRNA sequencing data and found a positive correlation between *LNC6302* and *SLC22A16* expression during chicken adipocyte differentiation [7], with *LNC6302* being located adjacent to *SLC22A16*. The mechanism of action of lncRNA is correlated with its expression distribution, and there are different regulatory mechanisms in the nucleus or cytoplasm [59,60,61]. RNA FISH indicates that *LNC6302* is expressed in both the nucleus and cytoplasm, suggesting that the mechanisms by which *LNC6302* regulates chicken adipocyte differentiation may be diverse. One of the mechanisms of action of lncRNA is the cis regulation of the expression of neighboring protein coding genes [62]. Studies have reported that lncRNA *AC092159*.2 can cis-regulate the transcription of *TMEM18* and thereby affect adipocyte differentiation [63]. The lncRNA *BADLNCR1* inhibits *GLRX5* transcription activity and inhibits bovine adipocyte differentiation [64]. Therefore, we focused on the relationship between *LNC6302* and *SLC22A16*. As shown in the results, the downregulation of *LNC6302* inhibited the expression of *SLC22A16*, and the overexpression of *SLC22A16* also restored adipocyte differentiation after the downregulation of *LNC6302*.

## 5. Conclusions

In summary, our data indicate that *SLC22A16* is a positive regulatory factor for the proliferation and differentiation of chicken adipocytes, and reveal that *LNC6302* promotes the differentiation of abdominal fat by regulating the expression of *SLC22A16* in chickens. This study enriches the regulatory network of chicken adipocyte differentiation, and it provides a potential target for molecular breeding.

## Data Availability

All RNA-seq datasets supporting the results of this article have been submitted to the National Center for Biotechnology Information (NCBI) Gene Expression Omnibus (GEO). The accession numbers are SRR7067303, SRR7067304, SRR7067305, SRR7067306, SRR6459507, SRR6459508, SRR6459509, and SRR6459510.

## Supplementary Materials

The following supporting information can be downloaded at: https://www.mdpi.com/article/10.3390/genes15060758/s1, Figure S1: The characteristic of chicken LNC6302 sequence, Table S1: Primer sequences for RT-qPCR.
