# Supplementary material for: Long Noncoding RNA 6302 Regulates Chicken Preadipocyte Differentiation by Targeting SLC22A16
Source: Genes (Basel). 2024 Jun 9;15(6):758. doi: 10.3390/genes15060758 (PMC11203196; doi:10.3390/genes15060758)
Supplement: Supplementary file 1 [file genes-15-00758-s001.zip › genes-2999311-supplementary.pdf]

Supplementary Table S1: Primer sequences for RT-qPCR.

	Gene name	Sequence	Accession NO.
qRT-PCR	*SLC22A16*	F: 5’- CTGGGTCACTTTGGCAGATTTC -3’<br>R: 5’- AGATGTCCTCCAGACTTGCT -3’	NM_001199197.1
	PPARG	F: 5’- ACCTCACGAGGAGTCTTCCA -3’<br>R: 5’- GCTTCTCCTTCTCCGCTTGT -3’	NM_205107.1
	CEBPA	F: 5’- CCTACGGCTACAGAGAGGCT-3’<br>R: 5’- TGATGTCGATGGAGTGCTCG-3’	NM_001031459.2
	FABP4	F: 5’- CTGCTACCTGGCCTGACAAA -3’<br>R: 5’- CAGTGTGCCACTGTCTAGGG -3’	XM_025152633.1
	LPL	F: 5’- ACTGAAACTTTTTCGCCGCT -3’<br>R: 5’- ATCTCAGCTTCGGGATCGGA -3’	XM_015276085.2
	β-actin	F: 5’-CAGCCAGCCATGGATGATGA-3’<br>R: 5’-ACCAACCATCACACCCTGAT-3’	NM_205518.1
	CCND1	F: 5’-ATAGTCGCCACTTGGATGCT-3’<br>R: 5’-AACCGGCTTTTCTTGAGGGG-3’	NM_205381.1
	PCNA	F: 5’-ACAATGCGGATACGTTGGCT-3’<br>R: 5’-CCAATGTGGCTGAGGTCTCT-3’	NM_204170.2
	CDK1	F: 5’-ACGTGATCCACACGGAGAAC-3’<br>R: 5’-GCAGCTGGAACAGGTAGCTC-3’	NM_205314.1
	LNC6302	F: 5’-GGAAGCATTGGAAAATGCCGT-3’<br>R: 5’-TCTGCACACCACCACCTTTC-3’	
PCR-CDS	*SLC22A16*	F: 5’-CCCAAGCTTATGGCTTCTGGCTCCGAGCAGCTCT-3’<br>F: 5’-CCGGAATTCCTTTCTCAGTGCTGACTGTTTCTTGACCAA-3’	

Supplementary Figure S1: The characteristic of chicken LNC6302 sequence.

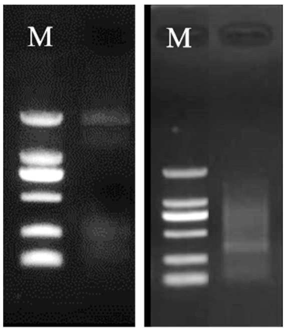

3'RACE 5'RACE

3'RACE-1

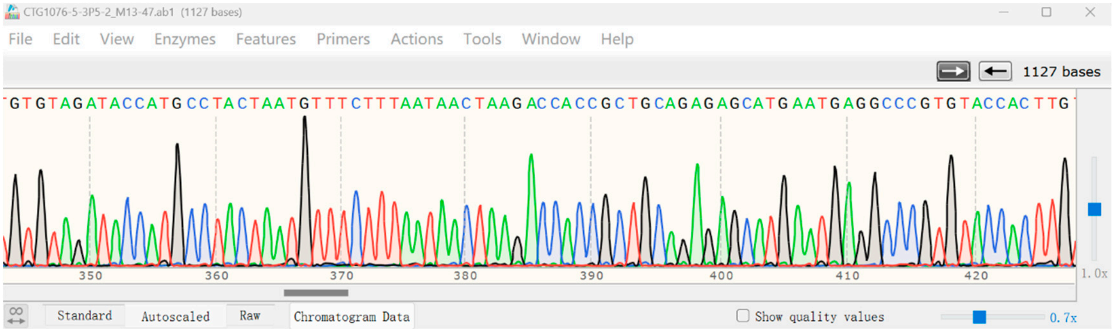

3'RACE-2

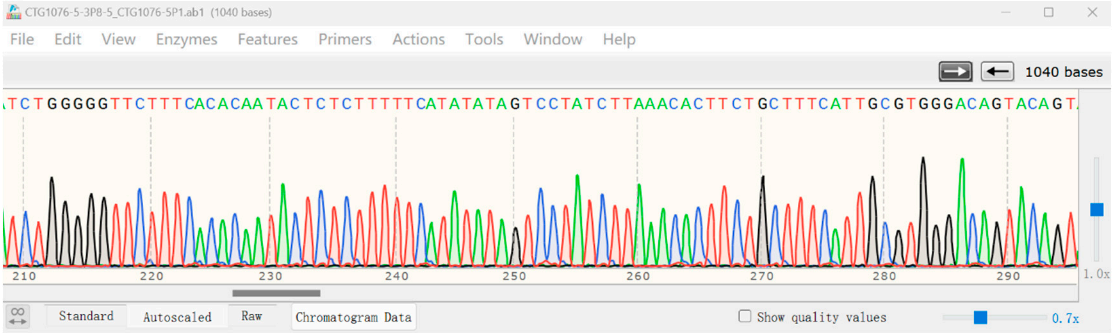

5'RACE-1

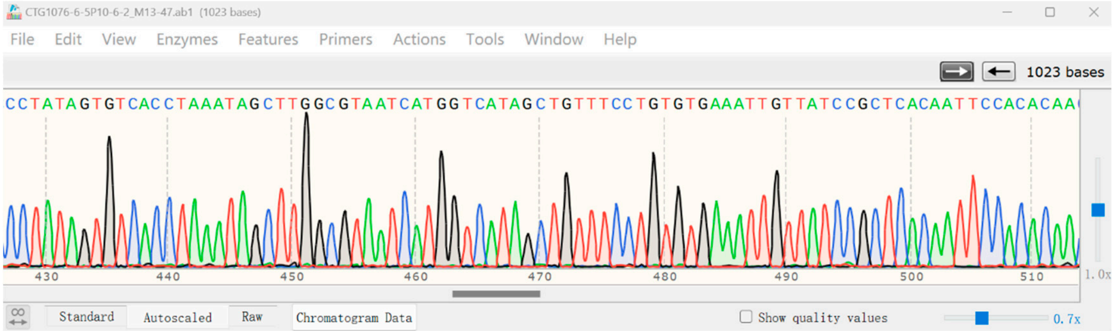

>NONGGAT006302.2(lnc6302)

CTAAAGACTTGTTTTGAATGAATGAAACTTCACTGGTAAGTTTGGATACTGT

CCCTGGAAAAAAAAAATACTGTTTGAAAAGTCACTTCATTAATATGCTGTG
CTTTAATTGTTTCGTATTCTGTCTTTCATAGGAAGCATTGGAAAATGCCGTGT
AGAGTGAGGTAGAAGAGAAACACAGGGAATTAGCCTGGGGCGCAGCAGC
TTTCGCCCTGAAAAAAAAGAGACACACAGTGATGGAATAGAGTAGAGAGC
TGAGATTAAAGGCAGGAAAGGTGGTGTGCAGATGTCCAGAATAGTGG
GAGTTCACAGGGCATAAGAGTTGCTTGTTGGAATCATGGCTCTTCTGAGTG
GAATATTAACATTAATGCTTCCAGAGACACTTGGAAAACCTTTGACTAACA
CTTTGGAGGAAGCTACAGAAATGGGAAGAAAGAAGGAAAACTACTCAGG
AAAGACTCCTCCAGCAGACAGTGGTGCAGCATTAGAAAAGATAGAGATGT
TTGGTCAAGAAACAGTCAGCACTGAGAAATAAAGCAAGAGCAAAATGGCC
GCTCCCAGTTTTAACGATTATATAATTTATAGGTCATTTTATTCAAGAAAAGA
CTGTTCTATAATATCTGTGCCTTTTAATTTGTAACACATCTTGCCTTTTTGAC
AGAGCAAGTGTATGAAACCTTTTCAACTTGACTGGGGTACTGCAGAAAATC
TGATGAGATTTCTAGATCCTCTCTCAAACTTATTTTGCCTTTAGAGAACCTG
TCTGACTTGCTGTGATTACTTTAAATTCTGTAACCTACCTGATTACATATGTG
TAAAAAAAAGTGCCACTTCTTACTTCAGATTTTCTGTTGTTAGACTTGTAAG
CCCACCACAGTGTGCCTCTTCCTTTTGCAGTCGTCGGTTACAGACTCGAGA
ATTCAATACGCAGTGAAGTCAAACTCACAGTTATAACTCAAGAGAGAAGCC
TTAGCGTTTCATCAGGCCACACTAATAATTTCAAACTTAAACTATTTTATTTT
TTTTTCCAAAACTGCTTCCAGAAAAGCTGTCATTTCTAACAAAAGTATTCTG
CCGAATGGCTTCTGATTGAAAAAACAATATTAATTTTGCAAACTGTGCGAAT
AGTTTGTATACTGTTCATTCTGTTGAATGGTAAATGTATTTTTTTAGTAGCTT
AGAAAAAATTGTTCACTGGCCCTGCTGCTGCCTTAGTAAGTTCTTTTTTAGA
GAAGAGTGCCTGATGCCTTTTAAAGTATTTTGCATTCCAGACCACTATGTCA
GTGCGCCCCATCTCGCTTTGTTCAGCTCACATTAAGTCTCAGATTTGCCTAT
TGCTTCAGAGCAACACCAGCAGCTTTGCCAAACACTTCCACGGAGGCTCA
GCGCTTCTTAGTCCTCAGAGGAACAGAACAAGTGGTACACGGGCCTCATTC
ATGCTCTCTGCAGCGGTGGTCTTAGTTATTAAAGAAACATTAGTAGGCATGG
TATCTACACACCATTCGTATCAGCCTCCATAGACTCATGTTGGCTCTGTAGCT
CCATTTTTTCCTTACAGTACAAAAAATCAGTTTCAAAAGTATGAATTAAAAA

AATGATATCTAGAGGACAGTGAGTGACCAGTTATTCTGGTATTTTTAGTCAA
AGTATCTATCAGTGTCTTTTAGATCTTGTGTGAGCATCAACACTCACTTACT
GGTGCAGTGACTGTGCCTTATGACTGTGGACCATAGAAAACTTGTAGATAG
GAAAACATAATTCTAGCTGCTCATTTGTGGCTTTCTGTCCCAGCTCTCTTTT
TTTCAGTCTACCTCCAATCCCAGAGGTTTCTTCCTTGCCTGTGAGTCTCTAT
CCCCTCAGCCACCCTTGTTTGAGCGGGCTGGCGTGGGAAGCTTCTGTACAT
CCCTTGCCTGGGTTGTGTGAGCCCTGCCACCCCTGGTGTGAAATCAGAGTT
CTTCTCACCTGAGGCACCTACCCACAGGCTAACTGACACAATCTCATCAAA
ACAAGTCTTTCCATGTTTACTTCTTTGACACGTACCCCAAAATCAAGACAC
CTAAATTATTGTAGATAAATATTCTACATGTTGTATAGTATATTTGTCATATTTT
TCTATTATATCTGCATGACCATATTAGTTTTAGTGAATTCTGAAAACAGAGTA
ATTATTTATATCCACCTATAAAATATTCTTTCTACTTCACTGTACTGTCCTAGA
GTGCCAAGTAGCACTTTGGTTCTGGGCATAGGATTCAGTCATGTCTTATGTG
ACAACTAATGTGCTTTGAAGGCAAGCAAAAATCAGTAAGTTTAAATTTAGG
GTGTTTGTGTGCAACAGTTAAAGAGGTCTTTGTTCGCCCTTCAGTTATGTG
GGTGTCCCAGTATTTGCAGGGAAAACTTCTGAAGAGAACTGCATGGATTAC
TATGGCTTCTATCAAGATATAATTTGCTCAGCAAAAGTATGTGCTTTTAACCT
TACCCATGTGAGTAATCCCATCATTATTCAGAGGAGCACTTGAAGACATACT
TTGATGACTAAAGATAGGATTACTCATATGCTGTGGCTTTCTGTTTTGCTCA
GGAACAGACCCAGTATTCTTCCACTGCTTTCAGATCCCCCTGTCCCAGTTTC
CGGAGCAGAACAGCTGCCCTTATCTGCAGGATCTGTTGCCAAACCCCGAGT
GCCCCACCTTAAACAAGGACTTAACCATACTAAGGAAGAAAATGGCATACA
GTAGTTCTAATGGGCAGTTAAACTGATCCAGTCTGACTTTACAGCCACTCTT
CCTTTCACAGGGCTCTAAATAATTGCATTATCTGGGGGTTCTTTCACACAAT
ACTCTCTTTTTCATATATAGTCCTATCTTAAACACTTCTGCTTTCATTGCGTG
GGACAGTACAGTATGTTTTAACGAGGTACTAAAGAACAATTTTGAATTAATT
TTTCTGCTTTCTTATCTGCAGAATATCTGATTTGGATATTACTCTGCTTTAAA
GGGTCAATGCATGTTATGTTTTCCATTGACTTGAGGGATTCAGGACCAGGTC
CTCCATAATTGGTATATGTAAGGTAGGGAAAGTAAGAAAAAAAGGAAAGAC
TTAAATGCACAATGATAGTGAGCAATCCTTGCAGGCTACAGTTTTAATCTGT

ACTCTGGAACACAATGCAGCCTGATGGCTTTTAACCCTTAAAGCCAGTTCT
TCCTGTCCCTGATGTCAGTTTGTACTTGGTCCTTAATTCAAGTGAGACTTGA
ATTTCCCTGAACCAATTATGCTGTGTTTTTTTCCCCTGGAAATAAAATATTAA
TTGATCAACAGGTACACCTTTTGGGATTTATGCATGAGTAGCTCTGAGGATG
CTGTGGGCTTTACTTTTTTACAGCAATGTGCTGCCTTTTAGCTGAGGTAGCA
CATTACTGTAAAAAAAAAAAAAAAGGGGGGGGGGGGGAAATGGTATTTCT
GAATTTCTACCTGTAGAATTTCTGTTTGTTTGCTTTCCCATGTGGAGAAAAA
TATGTTTAGCACTCGTTCTACCTTATAAATGTAGCACAGATGGGATGAGTTG
ATTTCTTTCTCTTCAGTATTCACAGATTATCTAAATCCTCTTGAAAGAGAGG
ACTGAGTTTGAGAACAAGAGCTTCTAAGTTGCTTTGGGGCATGTTTTCCTC
TCATTGCTAATAAAAACAATTCCTTTTCATAAAAAAAAAAAA
